# Five-partite entanglement generation between two optical frequency combs in a quasi-periodic *χ*^(2)^ nonlinear optical crystal

**DOI:** 10.1038/s41598-017-09346-3

**Published:** 2017-08-22

**Authors:** Guangqiang He, Yu Sun, Linxi Hu, Renhui Zhang, Xikun Chen, Jindong Wang

**Affiliations:** 1 State Key Laboratory of Advanced Optical Communication Systems and Networks, Electronic Engineering Department, Shanghai Jiao Tong University, Shanghai, 200240 China; 2 State Key Laboratory of Precision Spectroscopy, East China Normal University, Shanghai, 200062 China; 3 State Key Laboratory of Information Security, Institute of Information Engineering, Chinese Academy of Sciences, Beijing, 100093 China; 4 Guangdong Provincial Key Laboratory of Quantum Engineering and Quantum Materials, South China Normal University, Guangzhou, 510006 PR China

## Abstract

We theoretically prove five-partite entanglement can be produced among modes of two simultaneously generated optical frequency combs via second-order nonlinear interaction in a designed periodically poled lithium niobat (PPLN) crystal. An extendible model is proposed to analyze the entanglement characteristics of generated comb modes by applying van Loock and Furusawa criteria. Our proposal provides a potential approach for generating multipartite entangled states, the so-called cluster states, which are the key resources for quantum computation. Moreover, simultaneously generation of two entangled combs can provide much higher efficiency to generate cluster states.

## Introduction

Quantum entanglement is at the central part of applications such as quantum computation and quantum communication, such as quantum teleportation^1–3^, quantum key distribution^4^, quantum secure direct communication^5–7^, quantum machine learning^8^, and so on. To experimentally implement quantum computation, scalability is one of the most essential requirements^9^. Thus a so-called one-way quantum computer model^10^, in which quantum computation resource is provided by cluster states^11^, was proposed for the requirement. A cluster state is a multipartite entangled state in which any algorithm can be implemented by one-particle measurements only. Motivated by this discovery, a large number of works related to cluster states have been carried out^9, 12–20^. Various generation methods of cluster states are investigated, such as using beta-barium borate (BBO)^13^, cavity quantum electrodynamics (QED) techniques^15^, and optical frequency combs (OFCs)^9, 18–20^. Among these works, resorting to optical frequency combs is quite appealing. Some interesting works have been proposed about entanglement generated in optical combs. it is shown that optical-frequency combs are formed by the interaction between a cavity mode and a continuous-wave two-tone driving laser consisting of a pump field and a seed field via quantum dot-induced strong nonlinearity^21^. A large-scale quantum entanglement between two comb modes has very recently been explored in an interacting semiconductor quantum dot-photonic molecule system^22^.

OFCs have already found its way to applications such as precision spectroscopy, frequency transfer, astronomical spectral calibration, and generation of low-phase-noise microwave and radio frequency oscillators^23^. Initially, optical frequency combs were produced by mode-locked femtosecond laser^23^, it is stable but bulky and complex. For the sake of miniaturization, microresonators have been proposed and experimentally demonstrated to generate optical frequency combs based on the cascaded four-wave mixing (FWM), allowing considerable reduction of complexity, size and power consumption^24, 25^. FWM is third-order nonlinear effect and its conversion efficiency is much less than second-order nonlinear effects such as second-harmonic generation (SHG) and sum-frequency generation (SFG). Thus optical frequency combs generated via second-order nonlinearity have drawn growing attention. Iolanda *et al*.^26^ and Ville *et al*.^27^ have shown that two OFCs can be produced simultaneously by cascaded SHG and SFG. In their works, different features and generation regimes are investigated, good spectral quality is also shown. Inspired by their works, we proposed a novel model to analyze the entanglement characteristics of the two combs in this paper. Simultaneously generation of two entangled combs can provide much higher efficiency to generate cluster states, making an important step for quantum computation. Without loss of generality, we simplify the two combs with three modes in each comb, thus there are six modes in total.

The rest of this paper is arranged as follows. In result and discussion, system model and output fluctuation spectra are given as two parts. First, we describe the physical model for the generation of five-partite entanglement, then derive the main values and fluctuations of the output fields in system model. In output fluctuation spectra, the entanglement characteristics of the modes in two frequency combs are investigated. In method, we introduce the principle of designing the model and analyzing the four-partite entanglement.

## Results and Discussion

### System Model

**Figure 1:**
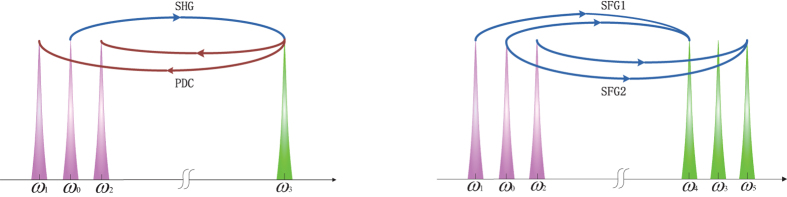
Second-harmonic generation (SHG) with cascaded down-conversion (PDC) process gives rise to the frequency components *ω*_3_, *ω*_1_ and *ω*_2_, which in turns leads to two sum-frequency processes (SFG1, SFG2) and generate another two subharmonic components *ω*_4_, *ω*_5_.

In this model, two output frequency combs consisted of six modes are generated by cascaded second-order nonlinear processes as shown in Fig. 1. First, a pump with frequency *ω*_0_ generates the beam with frequency *ω*_3_ through a second-harmonic generation. Then, two beams with frequency *ω*_1_ and *ω*_2_ are generated by a down-conversion process. Last, two beams with frequency *ω*_4_ and *ω*_5_ are generated by two sum-frequency processes.

According to the above nonlinear processes, The energy conversion and phase-matching conditions can be written as follows:

1 $$SHG:{\omega }_{0}+{\omega }_{0}={\omega }_{3},{k}_{0}+{k}_{0}+{G}_{1}={k}_{3},$$

2 $$PDC:{\omega }_{1}+{\omega }_{2}={\omega }_{3},{k}_{1}+{k}_{2}+{G}_{2}={k}_{3},$$

3 $$SFG1:{\omega }_{0}+{\omega }_{1}={\omega }_{4},{k}_{0}+{k}_{1}+{G}_{3}={k}_{4},$$

4 $$SFG2:{\omega }_{0}+{\omega }_{2}={\omega }_{5},{k}_{0}+{k}_{2}+{G}_{4}={k}_{5}\mathrm{.}$$

**Figure 2:**
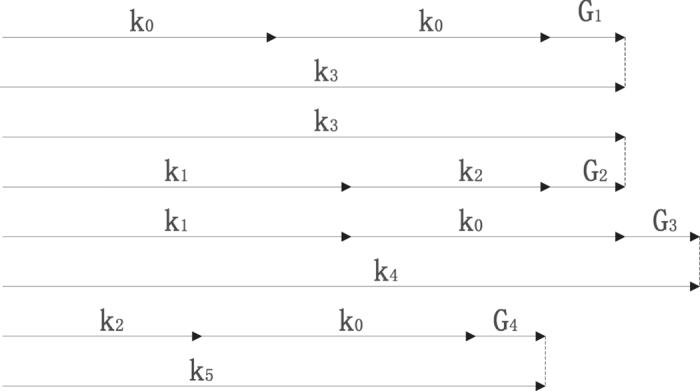
The quasi-phase matching scheme for the cascaded nonlinear processes.

*k*_*i*_(*i* = 0, 1, 2, 3, 4, 5) are the corresponding wave vectors of the six output modes with frequency *ω*_*i*_(*i* = 0, 1, 2, 3, 4, 5). *G*_1_, *G*_2_, *G*_3_, *G*_4_ are the four reciprocals needed to compensate the phase mismatching. The quasiphase-matching sketch is plotted in Fig. 2.

**Figure 3:**
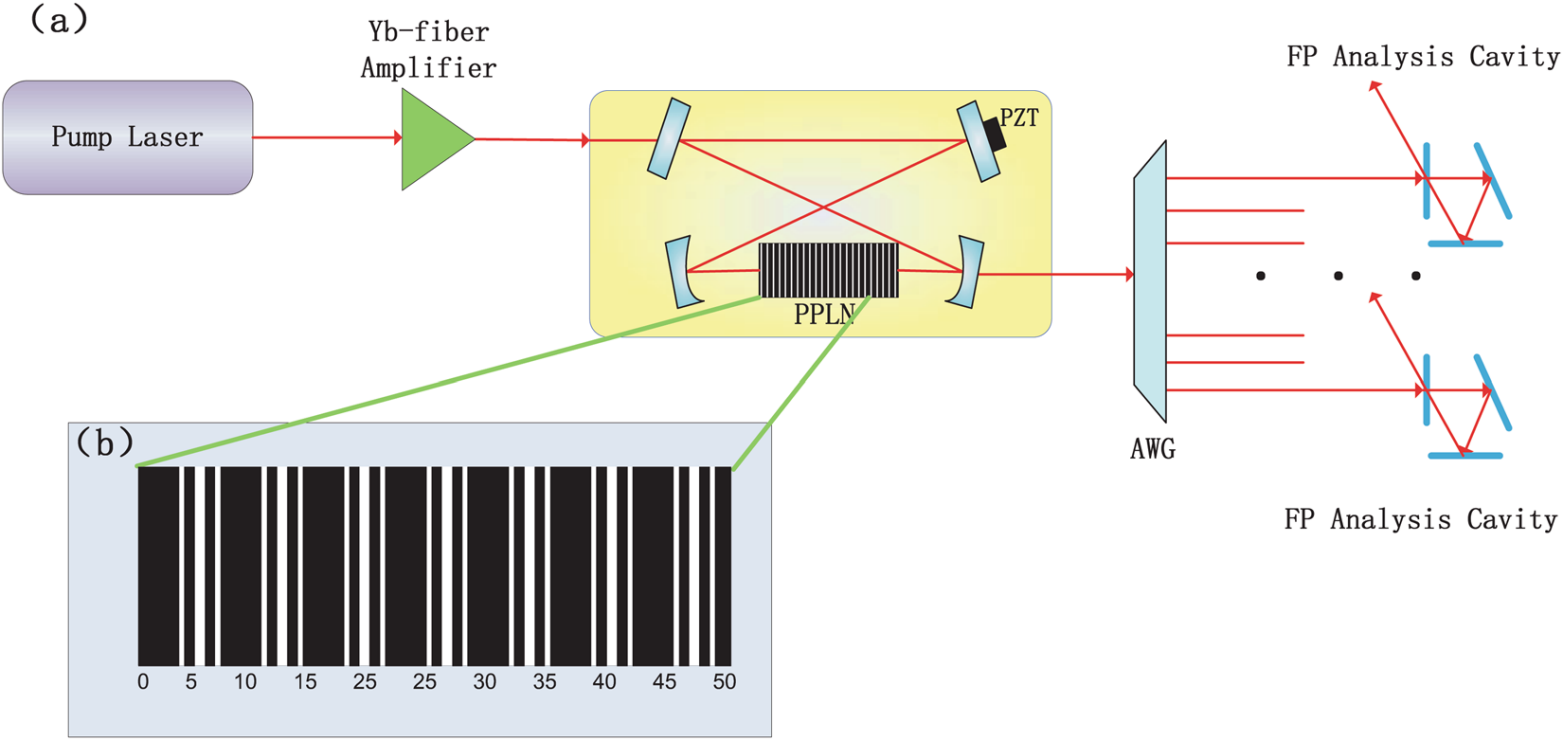
Experimental setup. (**a**) A four-mirrors travelling wave cavity, with a PPLN crystal, is pumped by an amplified cw laser. The nonlinear cavity output beams are separated by an arrayed waveguide grating (AWG) and then analyzed by the Fabry-Perot (FP) analysis cavities. (**b**) The structure of the PPLN OS.

To obtain the reciprocal-lattice vector for corresponding quasiphase mismatch, an optical superlattice (OS) is needed, and the superlattice can be designed using a dual-grid method. Due to Iolanda *et al*.’s work^26^, we set the six wavelengths with frequency *ω*_*i*_(*i* = 0, …, 5) at 1064.45, 1065.54, 1063.36, 532.225, 532.50, 531.95 nm, respectively, and the temperature is 39.5 °C. The tiling vectors of the OS are calculated to be 1.739, 1.740, 1.735, 1.742 *μm*. The structure of the PPLN OS is shown in Fig. 3(b), where the index of refraction of the black blocks is negative and the index of refraction of the white blocks is positive. The unit of length in Fig. 3b is micrometer.

Since the structure of the PPLN crystal is obtained, we design a schematic diagram of physical system as shown in Fig. 3. The system is pumped by a continuous-wave pump laser at 1064.45 nm, amplified by a Yb:fiber amplifier. Then the pump beam enters the cavity through a plane coupling mirror, while other mirrors are high-reflectivity. The high-reflectivity plane mirror is mounted on a piezoelectric actuator (PZT) for cavity length control and the designed PPLN crystal is placed between the two curved mirrors. The nonlinear processes give rise to the output frequency combs, which will finally be separated by an arrayed waveguide grating (AWG) and analyzed by the Fabry-Perot (FP) analysis cavities.

Due to the nonlinear processes proposed above, the interaction Hamiltonian *H*_*i*_ for the nonlinear processes, the Hamiltonian for the pump beam *H*_*p*_ are as follow:

5 $${H}_{p}=i\hslash {a}_{0}^{\dagger }\varepsilon +H.c.,$$

6 $${H}_{i}=i\hslash ({\kappa }_{1}{a}_{0}{a}_{0}{a}_{3}^{\dagger }+{\kappa }_{2}{a}_{1}^{\dagger }{a}_{2}^{\dagger }{a}_{3}+{\kappa }_{3}{a}_{0}{a}_{1}{a}_{4}^{\dagger }+{\kappa }_{4}{a}_{0}{a}_{2}{a}_{5}^{\dagger })+H\mathrm{.}c\mathrm{.}$$

where *κ*_*i*_(*i* = 1, 2, 3, 4) are the dimensionless nonlinear coupling coefficients of the nonlinear processes, ${\hat{a}}_{i}$ are annihilation operators of the modes with frequency *ω*_*i*_.

An optical oscillator is an open system since it not only exhibits intrinsic scattering loss with a photon decay rate of *γ*_*k*0_ (for mode k), but also couples waves to the coupling waveguide with an external coupling rate of *γ*_*kc*_. In order to describe such an open system, we present the loss and out-coupling terms as:

7 $${L}_{k}\hat{\rho }={\gamma }_{k}\mathrm{(2}{a}_{k}\hat{\rho }{a}_{k}^{\dagger }-{a}_{k}^{\dagger }{a}_{k}\hat{\rho }-\hat{\rho }{a}_{k}^{\dagger }{a}_{k}).$$

where $\hat{\rho }$ stands for the density matrix of system and *γ*_*k*_ = *γ*_*kc*_ + *γ*_*k*0_ represents the damping rate of the loaded cavity. Then the output field is determined by the well-known input-output relation given as ref. 28

8 $${b}_{out}-{b}_{in}=\sqrt{\gamma }a\mathrm{.}$$

in which *b* is the boson annihilation operator for the bath field outside the cavity.

As for the system model presented previously, whole procedure could be governed by the following master equation:

9 $$\frac{\partial \hat{\rho }}{\partial t}=-\frac{i}{\hslash }[{H}_{p}+{H}_{i},\hat{\rho }]+\sum _{k=0}^{5}\,{L}_{k}\hat{\rho }\mathrm{.}$$

The above master equation can be converted into the equivalent *c*-number Fockker-Planck equation in *P* representation, which may be written as a completely equivalent stochastic differential equation:

10 $$\frac{\partial \alpha }{\partial t}=F+B\eta ,$$

where

11 $$\alpha =[{\alpha }_{0},{\alpha }_{1},{\alpha }_{2},{\alpha }_{3},{\alpha }_{4},{\alpha }_{5},{\alpha }_{0}^{\ast },{\alpha }_{1}^{\ast },{\alpha }_{1}^{\ast },{\alpha }_{3}^{\ast },{\alpha }_{4}^{\ast },{\alpha }_{5}^{\ast }],$$

12 $$\eta =[{\eta }_{1},{\eta }_{2},{\eta }_{3},{\eta }_{4},{\eta }_{5},{\eta }_{6},{\eta }_{7},{\eta }_{8},{\eta }_{9},{\eta }_{10},{\eta }_{11},{\eta }_{12}],$$

13 $$F=[\begin{array}{c}f\\ {f}^{\ast }\end{array}],$$

14 $$f=(\begin{array}{c}{\gamma }_{0}{\alpha }_{0}-\varepsilon +2{\kappa }_{1}{\alpha }_{0}^{\ast }{\alpha }_{3}+{v}_{3}{\alpha }_{1}^{\ast }{\alpha }_{4}+{\kappa }_{4}{\alpha }_{2}^{\ast }{\alpha }_{5}\\ {\gamma }_{1}{\alpha }_{1}-{\kappa }_{2}{\alpha }_{2}^{\ast }{\alpha }_{3}+{\kappa }_{3}{\alpha }_{0}^{\ast }{\alpha }_{4}\\ {\gamma }_{2}{\alpha }_{2}-{\kappa }_{2}{\alpha }_{1}^{\ast }{\alpha }_{3}+{\kappa }_{4}{\alpha }_{0}^{\ast }{\alpha }_{5}\\ {\gamma }_{3}{\alpha }_{3}-{\kappa }_{1}{\alpha }_{0}{\alpha }_{0}+{\kappa }_{2}{\alpha }_{1}{\alpha }_{2}\\ {\gamma }_{4}{\alpha }_{4}-{\kappa }_{3}{\alpha }_{0}{\alpha }_{1}\\ {\gamma }_{5}{\alpha }_{5}-{\kappa }_{4}{\alpha }_{0}{\alpha }_{2}\end{array}).$$

${\alpha }_{i}={\bar{\alpha }}_{i}+\delta {\alpha }_{i}$, where *α*_*i*_ are the fields with frequency *ω*_*i*_, ${\bar{\alpha }}_{i}$ are mean values of *α*_*i*_, and *δα*_*i*_ are the fluctuations of the fields. *η*_*i*_(*i* = 1, … 12) are the real noise terms. Matrix *B* could be obtained by the relationship *D* = *BB* ^*T*^. *D* matrix we introduced here stands for the diffusion matrix, which is given by

15 $$D=(\begin{array}{cc}d & 0\\ 0 & {d}^{\ast }\end{array}),$$

where *d* is given by

16 $$d=(\begin{array}{cccccc}-2{\kappa }_{1}{\alpha }_{3} & -{\kappa }_{3}{\alpha }_{4} & -{\kappa }_{4}{\alpha }_{5} & 0 & 0 & 0\\ -{\kappa }_{3}{\alpha }_{4} & 0 & {\kappa }_{2}{\alpha }_{3} & 0 & 0 & 0\\ -{\kappa }_{3}{\alpha }_{5} & {\kappa }_{2}{\alpha }_{3} & 0 & 0 & 0 & 0\\ 0 & 0 & 0 & 0 & 0 & 0\\ 0 & 0 & 0 & 0 & 0 & 0\\ 0 & 0 & 0 & 0 & 0 & 0\end{array})\mathrm{.}$$

In order to obtain the steady-state solutions of the above processes, the noise terms and all the fluctuations can be neglected; thus the equations for the mean values of the fields can be written as:

17 $$\frac{\partial \delta {\bar{\alpha }}_{0}}{\partial t}={\gamma }_{0}{\bar{\alpha }}_{0}-\varepsilon +2{\kappa }_{1}{\bar{\alpha }}_{0}^{\ast }{\bar{\alpha }}_{3}+{\kappa }_{3}{\bar{\alpha }}_{1}^{\ast }{\bar{\alpha }}_{4}+{\kappa }_{4}{\bar{\alpha }}_{2}^{\ast },$$

18 $$\frac{\partial \delta {\bar{\alpha }}_{1}}{\partial t}={\gamma }_{1}{\bar{\alpha }}_{1}-{\kappa }_{2}{\bar{\alpha }}_{2}^{\ast }{\bar{\alpha }}_{3}+{\kappa }_{3}{\bar{\alpha }}_{0}^{\ast }{\bar{\alpha }}_{4},$$

19 $$\frac{\partial \delta {\bar{\alpha }}_{2}}{\partial t}={\gamma }_{2}{\bar{\alpha }}_{2}-{\kappa }_{2}{\bar{\alpha }}_{1}^{\ast }{\bar{\alpha }}_{3}+{\kappa }_{4}{\bar{\alpha }}_{0}^{\ast }{\bar{\alpha }}_{5},$$

20 $$\frac{\partial \delta {\bar{\alpha }}_{3}}{\partial t}={\gamma }_{3}{\bar{\alpha }}_{3}-{\kappa }_{1}{\bar{\alpha }}_{0}{\bar{\alpha }}_{0}+{\kappa }_{2}{\bar{\alpha }}_{1}{\bar{\alpha }}_{2},$$

21 $$\frac{\partial \delta {\bar{\alpha }}_{4}}{\partial t}={\gamma }_{4}{\bar{\alpha }}_{4}-{\kappa }_{3}{\bar{\alpha }}_{0}{\bar{\alpha }}_{1},$$

22 $$\frac{\partial \delta {\bar{\alpha }}_{5}}{\partial t}={\gamma }_{5}{\bar{\alpha }}_{5}-{\kappa }_{4}{\bar{\alpha }}_{0}{\bar{\alpha }}_{2}\mathrm{.}$$

Firstly we get the steady-state solution by setting the $\tfrac{\partial \delta {\bar{\alpha }}_{i}}{\partial t}=0$. Though the pump threshold can be obtained in analytical solution, it is too complex. So we choose to get the pump threshold by a numerical method. For example, when *γ*_0_ = 0.01, *γ*_1_ = 0.01, *γ*_2_ = 0.01, *γ*_3_ = 0.01, *γ*_4_ = 0.01, *γ*_5_ = 0.01, *κ*_1_ = 0.03, *κ*_2_ = 0.03, *κ*_3_ = 0.01, *κ*_4_ = 0.01, the threshold is *ε*_*th*_ = 0.003243. Notice that, when the pump wave power is below the threshold, there would be no steady solution for output waves. Thus we only investigate the entanglement characteristics above the threshold. Since the mean values of the fields in the cavity are obtained, they can be used to linearize the classical motion equations for the fields in the cavity to obtain the equations of the fluctuations of the fields:

23 $$\frac{\partial \delta \tilde{\alpha }}{\partial t}=M\delta \tilde{\alpha }+B\eta ,$$

in which $\delta \tilde{\alpha }={[\delta {\alpha }_{0},\delta {\alpha }_{1},\delta {\alpha }_{2},\delta {\alpha }_{3},\delta {\alpha }_{4},\delta {\alpha }_{5},\delta {\alpha }_{0}^{\ast },\delta {\alpha }_{1}^{\ast },\delta {\alpha }_{2}^{\ast },\delta {\alpha }_{3}^{\ast },\delta {\alpha }_{4}^{\ast },\delta {\alpha }_{5}^{\ast }]}^{T}$. M is the drift matrix given by

24 $$M=(\begin{array}{cc}{m}_{1} & {m}_{2}\\ {m}_{2}^{\ast } & {m}_{1}^{\ast }\end{array}),$$

where *m*_1_ and *m*_2_ is

25 $${m}_{1}=(\begin{array}{cccccc}-{\gamma }_{0} & 0 & 0 & -2{\kappa }_{1}{\bar{\alpha }}_{0}^{\ast } & -{\kappa }_{3}{\bar{\alpha }}_{1}^{\ast } & -{\kappa }_{4}{\bar{\alpha }}_{2}^{\ast }\\ 0 & -{\gamma }_{1} & 0 & {\kappa }_{2}{\bar{\alpha }}_{2}^{\ast } & -{\kappa }_{3}{\bar{\alpha }}_{0}^{\ast } & 0\\ 0 & 0 & -{\gamma }_{2} & {\kappa }_{2}{\bar{\alpha }}_{1}^{\ast } & 0 & -{\kappa }_{4}{\bar{\alpha }}_{0}^{\ast }\\ 2{\kappa }_{1}{\bar{\alpha }}_{0} & -{\kappa }_{2}{\bar{\alpha }}_{2} & -{\kappa }_{2}{\bar{\alpha }}_{1} & -{\gamma }_{3} & 0 & 0\\ {\kappa }_{3}{\bar{\alpha }}_{1} & {\kappa }_{3}{\bar{\alpha }}_{0} & 0 & 0 & -{\gamma }_{4} & 0\\ {\kappa }_{4}{\bar{\alpha }}_{2} & 0 & {\kappa }_{4}{\bar{\alpha }}_{0} & 0 & 0 & -{\gamma }_{5}\end{array}),$$

26 $${m}_{2}=(\begin{array}{cccccc}-2{\kappa }_{1}{\bar{\alpha }}_{3} & -{\kappa }_{3}{\bar{\alpha }}_{4} & -{\kappa }_{4}{\bar{\alpha }}_{5} & 0 & 0 & 0\\ -{\kappa }_{3}{\bar{\alpha }}_{4} & 0 & {\kappa }_{2}{\bar{\alpha }}_{3} & 0 & 0 & 0\\ -{\kappa }_{4}{\bar{\alpha }}_{5} & {\kappa }_{2}{\bar{\alpha }}_{3} & 0 & 0 & 0 & 0\\ 0 & 0 & 0 & 0 & 0 & 0\\ 0 & 0 & 0 & 0 & 0 & 0\\ 0 & 0 & 0 & 0 & 0 & 0\end{array})\mathrm{.}$$

For the validity of linearised quantum-fluctuation analysis, the quantum-fluctuation must be small enough compared with mean values. If the requirement that the real parts of the eigenvalues of −*M* stay non-negative is satisfied, the fluctuation equations will describe an Ornstein-Uhlenbeck process^29^, for which the intracavity spectral correlation matrix is given by

27 $$S(\omega )={(-M+i\omega I)}^{-1}D\,{(-{M}^{T}-i\omega I)}^{-1}\mathrm{.}$$

We introduce the quadrature operators for each mode in order to discuss the five-partite entanglement:

28 $${X}_{k}={a}_{k}+{a}_{k}^{\dagger },$$

29 $${Y}_{k}=-i({a}_{k}-{a}_{k}^{\dagger }),$$

with a commutation relationship of [*X*_*k*_, *Y*_*k*_] = 2*i*. Thus we know that *V*(*X*_*k*_) ≤ 1 could stands for the squeezed state based on our operator definition. *V*(*A*) = 〈*A* ^2^〉 − 〈*A*〉^2^ indicates the variance of operator *A*.

The output fields is determined by the well-known input-output relations Eq. 8. In particular, the spectral variances and covariances have the general form

30 $${S}_{{X}_{i}}^{out}(\omega )=1+2{\gamma }_{c}{S}_{{X}_{i}}(\omega ),$$

31 $${S}_{{X}_{i},{X}_{j}}^{out}(\omega )=2{\gamma }_{c}{S}_{{X}_{i},{X}_{j}}(\omega ),$$

*Y* quadratures have the similar expressions.

Multipartite entanglement criteria is given by the Van Loock and Furusawa (VLF)^30^. In our discussion, we consider Fokker-Planck equation in P representation and then analyse the entanglement condition that van Loock and Furusawa criteria are violated simultaneously. By using the above quadrature definitions, the five-partite criteria is given by

32 $${S}_{01}=V({X}_{0}-{X}_{1})+V({Y}_{0}+{Y}_{1}+{g}_{2}{Y}_{2}+{g}_{3}{Y}_{3}+{g}_{4}{Y}_{4}+{g}_{5}{Y}_{5})\ge \mathrm{4,}$$

33 $${S}_{12}=V({X}_{1}-{X}_{2})+V({g}_{0}{Y}_{0}+{Y}_{1}+{Y}_{2}+{g}_{3}{Y}_{3}+{g}_{4}{Y}_{4}+{g}_{5}{Y}_{5})\ge \mathrm{4,}$$

34 $${S}_{14}=V({X}_{1}-{X}_{4})+V({g}_{0}{Y}_{0}+{Y}_{1}+{g}_{2}{Y}_{2}+{g}_{3}{Y}_{3}+{Y}_{4}+{g}_{5}{Y}_{5})\ge \mathrm{4,}$$

35 $${S}_{25}=V({X}_{2}-{X}_{5})+V({g}_{0}{Y}_{0}+{g}_{1}{Y}_{1}+{Y}_{2}+{g}_{3}{Y}_{3}+{g}_{4}{Y}_{4}+{Y}_{5})\ge 4.$$

in which *g*_*k*_(*k* = 0, …, 5) are arbitrary real parameters that are used to optimize the violation of these inequalities. Considering the frequency component of *ω*_3_ is generated by SHG, it is hardly to entangle with other modes, we only investigate entanglement characteristics among other five mode. According to the symmetry between *ω*_1_ and *ω*_2_, we choose to investigate *S*_01_, *S*_12_ and *S*_14_ in our rest analysis.

### Output Fluctuation Spectra

According to Eqs [17–22], the stable solution is completely determined by three parameters: the total damping rate *γ*, the coupling coefficient *κ*, and the pumping power *ε*, which in turn determines the drift matrix M, the diffusion matrix D, and the intracavity spectral correlation matrix S. In addition, we also conclude that the parameter *γ*_*c*_ plays a role in the spectral correlation matrices according to Eqs [30 and 31]. In the following work, we will vary these parameters to investigate the entanglement.

**Figure 4:**
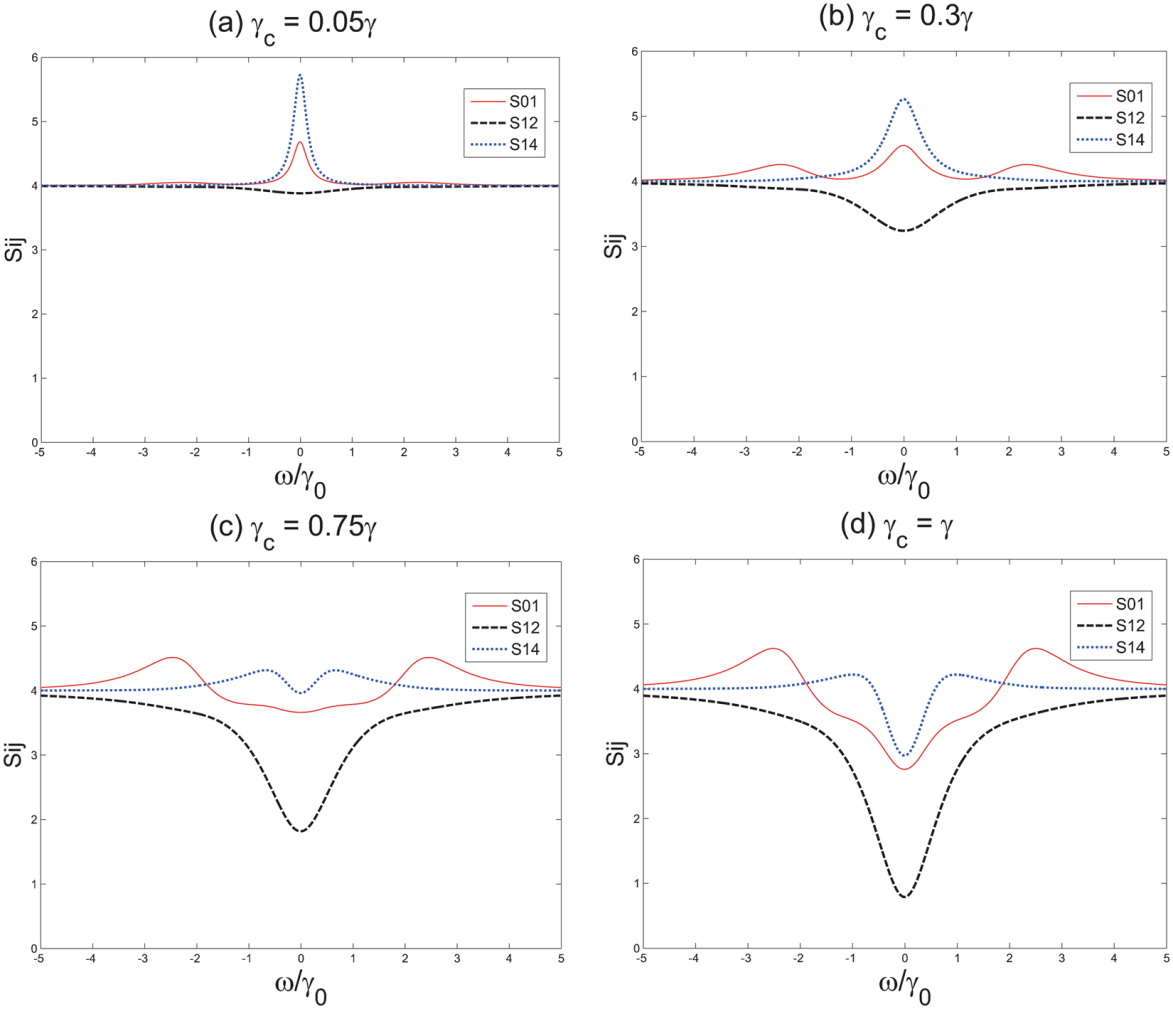
Four variances versus frequency of pump plots when *γ*_*c*_/*γ* is 0.05, 0.3, 0.7, 1. The pump power is fixed at 1.5*ε*_*th*_.

To begin with, we fix *κ*, *γ* and *ε* - the three parameters that governs the evolution in the cavity, and vary the *γ*_*c*_/*γ* ratio to investigate its influence on the entanglement. To satisfy the validly of the linearization method and calculate the quantum correlation spectra, the parameters should be chosen properly. Therefore, we set *γ*_0_ = 0.01, *γ*_1_ = 0.003, *γ*_2_ = 0.003, *γ*_3_ = 0.01, *γ*_4_ = 0.01, *γ*_5_ = 0.01, *κ*_1_ = 0.03, *κ*_2_ = 0.03, *κ*_3_ = 0.01, *κ*_4_ = 0.01. With above parameters, we plot the minimum of the variances versus the analysis frequency normalized to *γ* in Fig. 4 when *γ*_*c*_ takes a portion of 0.05, 0.3, 0.75 and 1.

From Fig. 4, we can see that when *γ*_*c*_/*γ* = 0.05, there is no entanglement between any two frequency modes. As we increase the out-coupling coefficient, the three frequencies in the first comb begin to entangle with each other. When *γ*_*c*_/*γ* = 0.75, the *ω*_1_ and *ω*_2_, which are respectively in two different combs, begin to entangle. And eventually when we set the portion to the *γ*_*c*_/*γ* = 1, the degree of entanglement is the largest compared with other case. Thus, we conclude that the entanglement among output modes increases as the radio *γ*_*c*_/*γ* increases. This can be explained that the higher portion the coupling coefficient takes, the less consumed entangled pairs are wasted in the internal loss.

**Figure 5:**
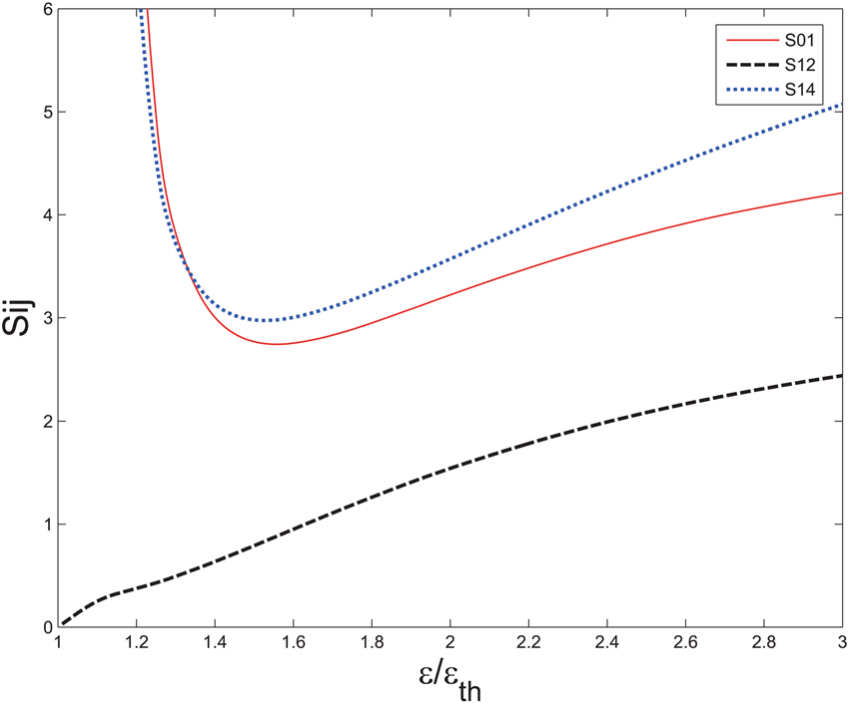
The minimum variance as a function of pump power. The external coupling coefficient is fixed at *γ*_*c*_ = *γ*.

**Figure 6:**
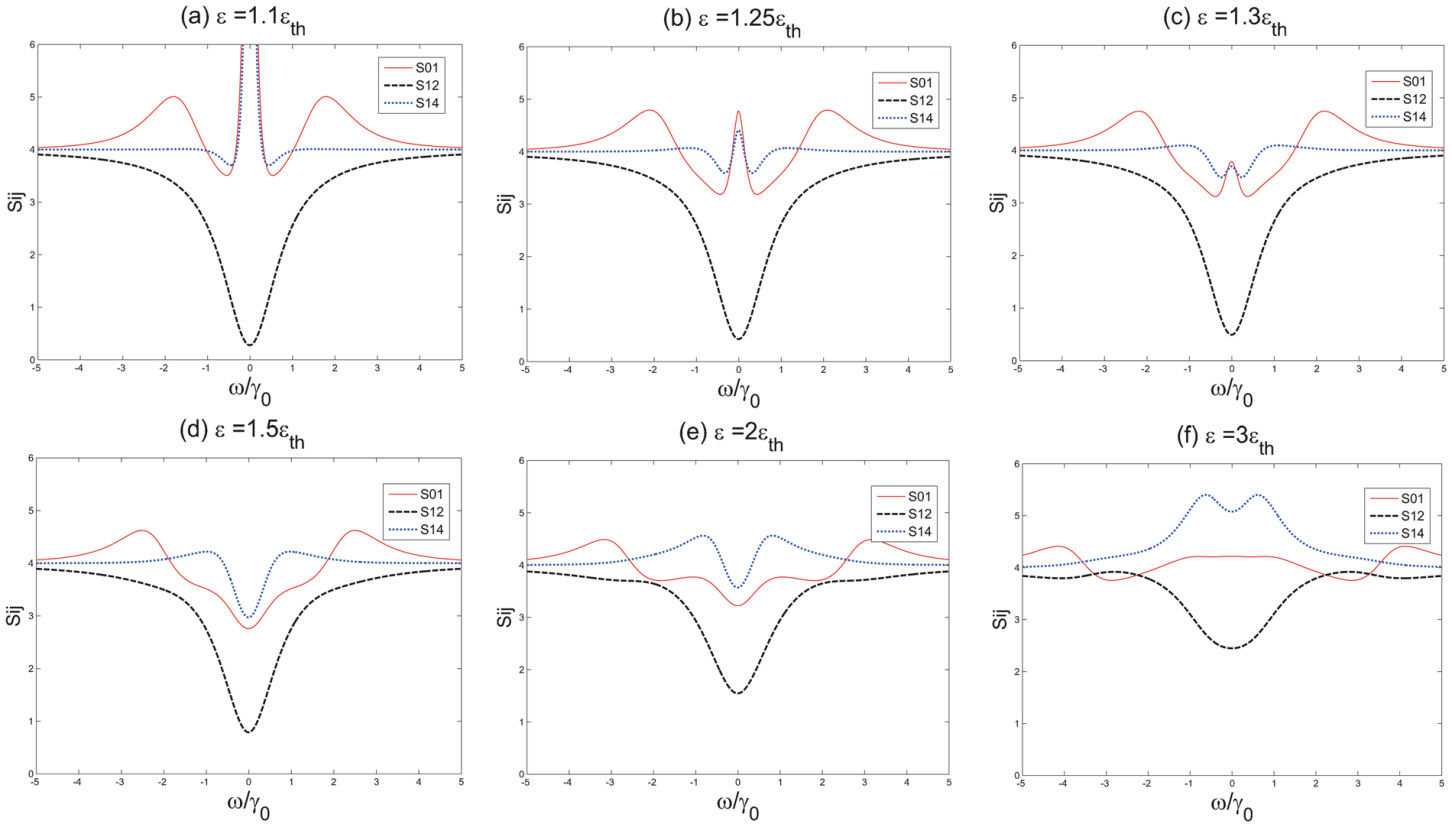
Extracavity variance versus frequency of pump power. The external coupling coefficient is fixed at: *γ*_*c*_ = *γ*.

In order to investigate the effect the pump power brings to the degree of entanglement, we firstly set the *γ*_0_ = 0 which means no intracavity loss in this part of discussion. With our previous discussion, the variance *S*_*i*_ as a function of *ω*/*γ* is merely determined by the parameter *ε*/*ε*_*th*_ while choose proper *κ* and *γ*. We plot the minimum variance throughout the noise power spectrum as a function of the pump power which has been normalized by *ε*_*th*_ in Fig. 5. We plot the minimum variance versus frequency under different pumping power in Fig. 6.

It can be inferred from Fig. 5 that the variance of *S*_01_ and *S*_14_ would first decrease as the pump power increasing and reach their minimum values when *ε* = 1.5*ε*_*th*_ around. Then *S*_01_ and *S*_14_ would ascend with the pump power while *S*_12_ increases as the pump power since the beginning. Considering that *S*_01_ and *S*_14_ are the short slabs of the entanglement model, we conclude that the 1.5*ε*_*th*_ is the best pump power in our case. That explains why we choose *ε* = 1.5*ε*_*th*_ in previous investigation.

In conclusion, we propose the theoretical model for the five-entanglement among modes of two optical frequency combs. By solving Fokker-Planck equation in P representation, we analysed the entanglement case where Van Loock and Furusawa criteria are violated. We analytically find that the intensity of entanglement is completed influenced by the *ε*/*ε*_*th*_, *ω*/*γ*, and *γ*_*c*_/*γ*. The results would offer a new path for the future study for entanglement over optical frequency combs generated via second-order nonlinear interaction.

## Method

We design the PPLN crystal using the so-called generalized dual grid method (DGM), which will phase match the four nonlinear processes. In this method, a dual structure, called dual grid, which contains all the topological information required to built the quasi-crystal is constructed and transformed to a quasi-crystal.

We analyze the entanglement case based on the Van Loock and Furusawa criteria. First, we obtain the main equation and transform it into Fokker-Planck equation. After we get the steady-solution of the above equations, we can obtain the equations of the fluctuations of the fields. Then, we calculate the spectral variances and covariances which will be applied into the Van Loock and Furusawa criteria. Finally, the influences of *ε*/*ε*_*th*_, *ω*/*γ*, and *γ*_*c*_/*γ* on the intensity of entanglement are analysed.

Our analysis method is also suitable for other types of entanglement, such as multi-partite GHZ state and logic-qubit entanglement, as long as the nonlinear processes are given we can derive the Hamiltonian expressions, then we can analyze the entanglement characteristics according to the method we propose above.
